# The Impact of Artificial Intelligence CNN Based Denoising on FDG PET Radiomics

**DOI:** 10.3389/fonc.2021.692973

**Published:** 2021-08-24

**Authors:** Cyril Jaudet, Kathleen Weyts, Alexis Lechervy, Alain Batalla, Stéphane Bardet, Aurélien Corroyer-Dulmont

**Affiliations:** ^1^Medical Physics Department, CLCC François Baclesse, Caen, France; ^2^Nuclear Medicine Department, CLCC François Baclesse, Caen, France; ^3^UMR GREYC, Normandie Univ, UNICAEN, ENSICAEN, CNRS, Caen, France; ^4^Normandie Univ, UNICAEN, CEA, CNRS, ISTCT/CERVOxy group, GIP CYCERON, Caen, France

**Keywords:** denoising, AI, PET, radiomics, medical imaging, convolutional neural network, VEREOS

## Abstract

**Background:**

With a constantly increasing number of diagnostic images performed each year, Artificial Intelligence (AI) denoising methods offer an opportunity to respond to the growing demand. However, it may affect information in the image in an unknown manner. This study quantifies the effect of AI-based denoising on FDG PET textural information in comparison to a convolution with a standard gaussian postfilter (EARL1).

**Methods:**

The study was carried out on 113 patients who underwent a digital FDG PET/CT (VEREOS, Philips Healthcare). 101 FDG avid lesions were segmented semi-automatically by a nuclear medicine physician. VOIs in the liver and lung as reference organs were contoured. PET textural features were extracted with pyradiomics. Texture features from AI denoised and EARL1 *versus* original PET images were compared with a Concordance Correlation Coefficient (CCC). Features with CCC values ≥ 0.85 threshold were considered concordant. Scatter plots of variable pairs with R2 coefficients of the more relevant features were computed. A Wilcoxon signed rank test to compare the absolute values between AI denoised and original images was performed.

**Results:**

The ratio of concordant features was 90/104 (86.5%) in AI denoised *versus* 46/104 (44.2%) with EARL1 denoising. In the reference organs, the concordant ratio for AI and EARL1 denoised images was low, respectively 12/104 (11.5%) and 7/104 (6.7%) in the liver, 26/104 (25%) and 24/104 (23.1%) in the lung. SUVpeak was stable after the application of both algorithms in comparison to SUVmax. Scatter plots of variable pairs showed that AI filtering affected more lower *versus* high intensity regions unlike EARL1 gaussian post filters, affecting both in a similar way. In lesions, the majority of texture features 79/100 (79%) were significantly (p<0.05) different between AI denoised and original PET images.

**Conclusions:**

Applying an AI-based denoising on FDG PET images maintains most of the lesion’s texture information in contrast to EARL1-compatible Gaussian filter. Predictive features of a trained model could be thus the same, however with an adapted threshold. Artificial intelligence based denoising in PET is a very promising approach as it adapts the denoising in function of the tissue type, preserving information where it should.

## Introduction

Imaging modalities are nowadays an essential diagnostic tool in medicine. From 2009 to 2019 the number of exams in the USA has increased by about 18%, 42% and 105% for CT, MRI and PET respectively (1). This increasing demand has exceeded the actual offer leading to unreasonable delay, weeks or even months for MRI and PET scans in France/Europe (2). An appropriate image denoising may help to reduce scanning time or even reduce injected dose for PET. It may allow to increase the number of examinations without impacting too much working hours or requiring the installation of new medical imaging devices. Deep learning as a subdivision of artificial intelligence (AI) allows to build promising denoising models.

We focused on PET imaging as it will benefit of denoising because of its long scanning time. Although many studies are actually investigating the clinical performance of this method, it may also impact other emerging fields such as imaging based predictive models, radiomics and other AI applications (3).

Medical images are basically a visual representation of different grey levels based on their density (CT), magnetic properties (MRI) or functional information (PET/SPECT). The distribution of the grey values characterizes the heterogeneity of the information. A fast-evolving field called radiomics provide a methodology to extract different features based on intensity, shape, texture from images in order to build predictive models (4). This approach holds great promises as being able to predict patient outcomes. They might allow personalized treatment. As an example, an overall survival predictive model including radiomics features was computed in lung cancer (5) This field is increasing with an annual growth rate of published papers of 177.82% between 2013 and 2018 (6). The models are very promising but there are still some efforts to be made to translate and implement them in a routine clinical setting (7).

Artificial intelligence is in the early phase of application in medical imaging. In this article, we used deep learning and more specifically convolutional neural network approaches which represent a subdivision of AI techniques. Today deep learning has a key role in image reconstruction, processing (denoising, segmentation), analysis and predictive modelling. These applications will develop even more in the future (8). In most of these tasks, they often outperformed a more traditional approach (9). A comparison of this type of AI based denoising algorithm on a PET/MR with clinical data show an increase of the contrast over noise ratio by 46.80 ± 25.23% compared to 18.16 ± 10.02% for a Gaussian filter only (10)]. Other methods studied in (10) like guided nonlocal means, block matching 4D or deep decoder improve the CNR oby24.35 ± 16.30%, 38.31 ± 20.26% and 41.67 ± 22.28% respectively. Denoising may also be performed during reconstruction, however this cannot be implemented on an existing machine. The most important limitation is the lack of FDA or CE certification of all those approaches. We focus our study on Subtle PET™ (Subtle Medical, Stanford, USA provided by Incepto, France). It is a post-processing FDA and CE approved denoising software for FDG PET  (11), based on convolutional neural networks (CNN), the most common deep learning architecture for image processing.

AI denoising and radiomics are two very promising fields in medical imaging. However, we are the first, to the best of our knowledge, to try to combine these two approaches for PET Imaging. We question whether a radiomics model using PET [^18^F] FDG trained on classical data is still valid after an AI denoising method. This study measured the stability of basic and radiomics PET features in lesions and normal reference organs when applying an AI denoising solution. We also wanted to provide an intuitive understanding on how images are affected by AI compared to a reference gaussian post filter routinely used in our center to generate EARL1 compatible PET series.

## Materials and Methods

This retrospective study was approved by the local institution review board. 113 patients referred to our oncological institution for an initial or follow-up [^18^F] FDG PET/CT exam between January and March 2020 were retrospectively included. We obtained an informed consent (non-opposition) from all patients. This observational study was in line with MR 004, a national French institution (INDS) defining health research conduct guidelines. The study population characteristics are shown in **Table 1**.

**Table 1:** Description of the patient cohort.

Patients (N)		133	Number
Sex		68%	female%
Age(Y)		61.5±13.5	mean±SD
		[24-89]	[range]
Weight (kg)		74±16	mean±SD
		[35-110]	[range]
BMI(kg/m2)		27±6	mean±SD
		[15-42]	[range]
Indication			
*Oncologic*	95 (84%)		Number (%)
	*Breast*	36 (32%)	
	*Lung*	17 (15%)	
	*Gynaecologic (except breast)*	14 (12%)		
	other malignancies (lymphoma, anal, colorectal, bladder, thyroid, head and neck, melanoma, myeloma) or mixed	28 (25%)	
*Diagnostic benign versus malignant*	14 (12%)		
*Miscellaneous*	5 (4%)		

Our PET center is accredited by EANM research limited (EARL) (12) and EANM imaging guidelines (13) were respected. The patients were injected with 4MBq/kg of [^18^F] FDG IV. PET images from skull base to mid-thighs were acquired on a digital PET/CT (VEREOS 2018, Philips Healthcare) during 1min/bed position. Once acquired, PET images were reconstructed with an 3D OSEM algorithm, 4 iterations, 4 subsets with point spread function (PSF) correction. Scatter and attenuation correction was applied. The spacing and matrix size were respectively of 2x2x2 mm^3^ and 288x288 pixels. An EARL1 reconstruction was also generated with the same parameters but convolved with a gaussian post filter of 7.2mm. CT scan parameters were 100-140 kV (BMI adaptive), with variable mAs according to an index dose right of 14 and an iterative reconstruction I dose 4; 64x0.625mm slice collimation, pitch of 0.83, rotation time 0.5 s, 3D modulation, matrix 512x512 and voxel size 0.97x0.97x 3 mm^3^. The PET mean dose was 5.32 mSv for a patient of 70 Kg. CT had a CTDI median value of 4.8 mGy and a DLP of 431.5 mGy.cm.

The originally reconstructed PET images (with PSF modelling) were denoised with a convolutional neural network (CNN) approach by a commercially available software, Subtle PET^®^ by Subtle Medical. SubtlePET™ uses a multi-slice 2.5D (5 slices) encoder-decoder U-Net DCNN to perform denoising. The software takes a low count PET image as the input and generates a high-quality PET image (close to full dose image) as the output. Accreditation from FDA and CE required robustness. The denoising model was trained on PET images from different centers and vendors. It employs a CNN-based method in a pixel’s neighborhood to reduce noise and increase image quality. Using a residual learning approach and optimized for quantitative (L1 norm) as well as structural similarity (SSIM), the software learns to separate and suppress the noise components while preserving and enhancing non-noise components. The images were directly sent from the PET console to a specific local server. Once transferred they were anonymized, denoised, deanonymized and pushed back to a clinical viewer. The mean treatment time was 45 s on a NVIDIA 1080 GPU processor.

All contours were performed in 3D slicer version 4.10 (14) on original PSF PET images and copied on AI denoised and EARL1 PET series. Spherical volumes of interest (VOI) were drawn in the reference organs: liver (3 cm radius, avoiding upper parts, tissue boundaries and major vessels) and lung (1.5 cm radius, drawn in the upper parts). Up to five FDG avid lesions per patient (including only the most intense ones), in total 101 lesions, were segmented by an experienced nuclear medicine physician. Segmented lesions consisted only of authentic malignant primary and metastatic lesions in solid tumors or lymphoma. A semi-automatic tool was employed to segment lesions. A VOI was created by clicking on the original PET image. This 3DSlicer module (PETTumorsSegmentation) is based on a highly automated optimal surface segmentation approach, which is a variant of the layered optimal graph image segmentation of multiple objects and surfaces segmentation (15). The VOI was than inspected and manually adjusted with a brush if needed. An automatic donut of 2 voxels diameters was grown around the lesion to calculate the lesion over background ratio. The mean analyzed metabolic volume was 20 (1-162) ml. The same VOI were used for original, AI denoised and EARL1like images.

The extraction of radiomics features was automatically carried out with the pyradiomics package (16) thus mostly complying with the Image Biomarker Standardisation Initiative (17). Images had a native isotropic spacing of 2x2x2 mm^3^ so an interpolation step was not necessary. As there is no consensus about the intensity discretization, a fixed bin number of 64 was used (18). A python code using simpleITK (19) was developed to extract all the radiomics features and is accessible in the supplementary information. Eight groups of radiomics features were computed. The intensity class contains first-order data, describing the distribution of voxel intensities within the image region defined by the VOI. They are commonly used and basic images metrics. The shape class is constituted of the 3D size and shape of the VOI. These shape features were excluded as the VOI was the same in all the images. A Grey Level Co-occurrence Matrix (GLCM) class describes the second-order joint probability function of an image region. Grey Level Size Zone Matrix (GLSZM) features quantify grey level zones in an image. A grey level zone is defined as the number of connected voxels that share the same grey level intensity (3D). The Grey Level Run Length Matrix (GLRLM) class testifies of grey level runs, which are defined as the length in number of pixels, of consecutive pixels that have the same grey level value (1D). Neighboring Grey Tone Difference Matrix (NGTDM) is a descriptor of the difference between a grey value and the average grey value of neighbors. A Grey Level Dependence Matrix (GLDM) characterizes the number of connected voxels within a distance from the center voxel in function of their grey level. Most features used in this study are in compliance with Imaging Biomarker Standardization Initiative (IBSI) (IBSI reference manual).IQ wavelets class contains two features, a local analyzing just the VOI and a global of the whole image. These metrics characterize image quality as the ratio between high and low wavelet frequencies.

The Concordance Correlation Coefficients (CCC) (20) were evaluated comparing the post processing IA denoised and EARL1 images to the original PET. CCC values of +/-1 describe a perfect positive/negative correlation and 0 no correlation. Features with a minimum CCC of 0.85 were considered as statistically reproducible and concordant (21). Scatter plots of variable pairs with R^2^ value was displayed for the coefficient of variation (CV) and mean SUV values to understand the difference of CCC’s in lesions and in liver when an AI denoising or EARL1 filter are used on original images. Mean SUV in lesions is presented using boxplots with minimum, maximum, 1st quartile and 3rd quartile to highlight the difference between the 3 series. A paired Wilcoxon signed rank test was used to compare features in original and AI denoised, and original and EARL1 images. P-values <0.05 were considered statistically significant. All the statistical analyzes were performed using python (22) and scipy.stats library. All the data and the python code of the analysis are available on https://github.com/AurelienCD/RadiomicsIA_PET_Depository_Manuscript-ID-692973.

## Results

**Figure 1:**
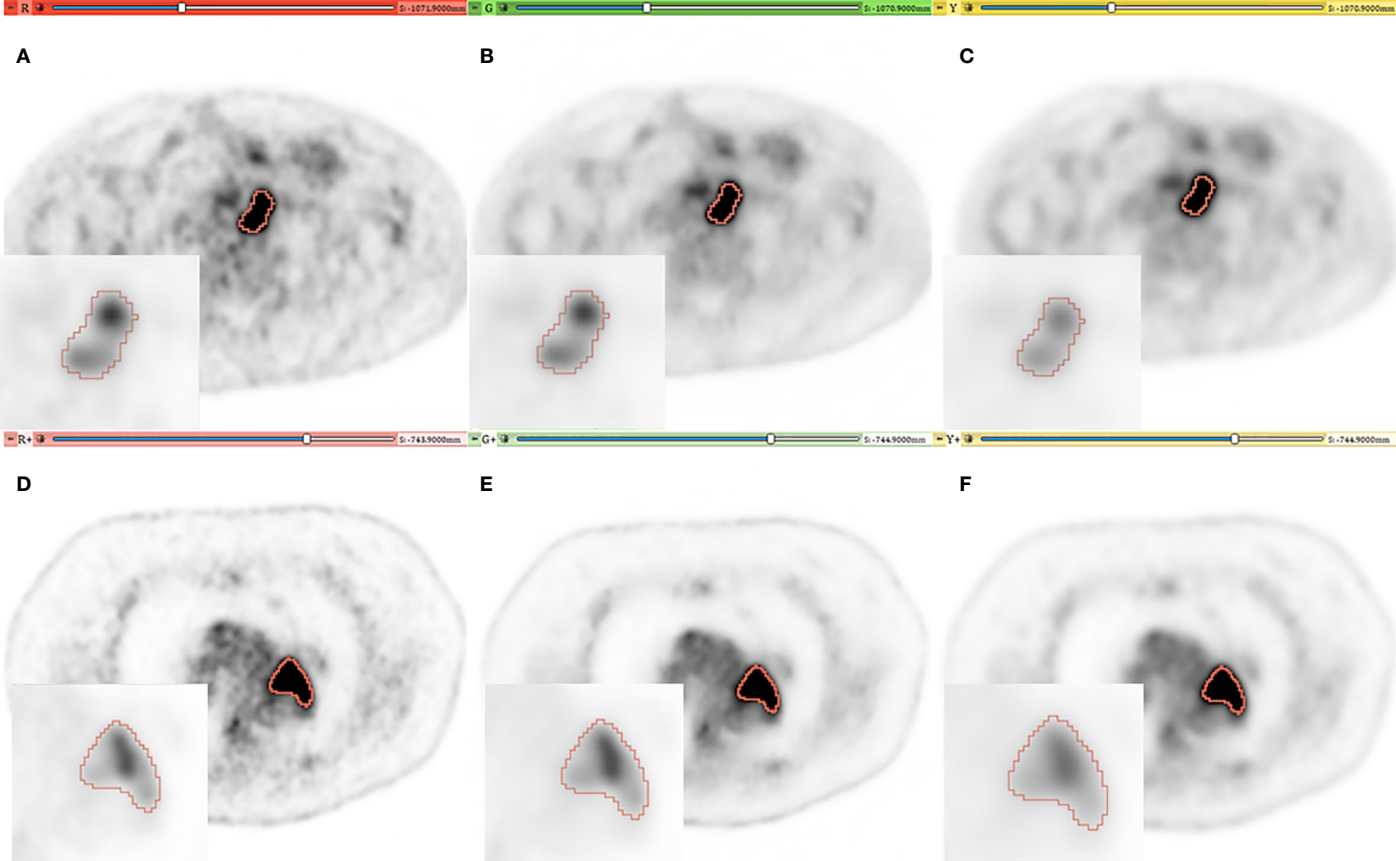
Representative PET imaging of two lesions in different patients with a SUV windowing of (0–5). **(A, D)** [red], **(B, E)** [green], **(C, F)** [yellow] for original, AI and EARL images respectively. A Zoom is added on each image with SUV windowing between (0–25) and (0–30) for the first and second patient.

A visual comparison of AI denoised (B) *versus* original images (A) shows that the AI approach seems to decrease noise in healthy tissues while preserving the intensity distribution in the lesion in **Figure 1**. In the EARL1-PET image (C) background noise is reduced, but also in the lesion the uptake intensity and distribution are affected. Similar observations can be deduced from the second patient’s images (**Figures 1D–F**).

**Figure 2:**
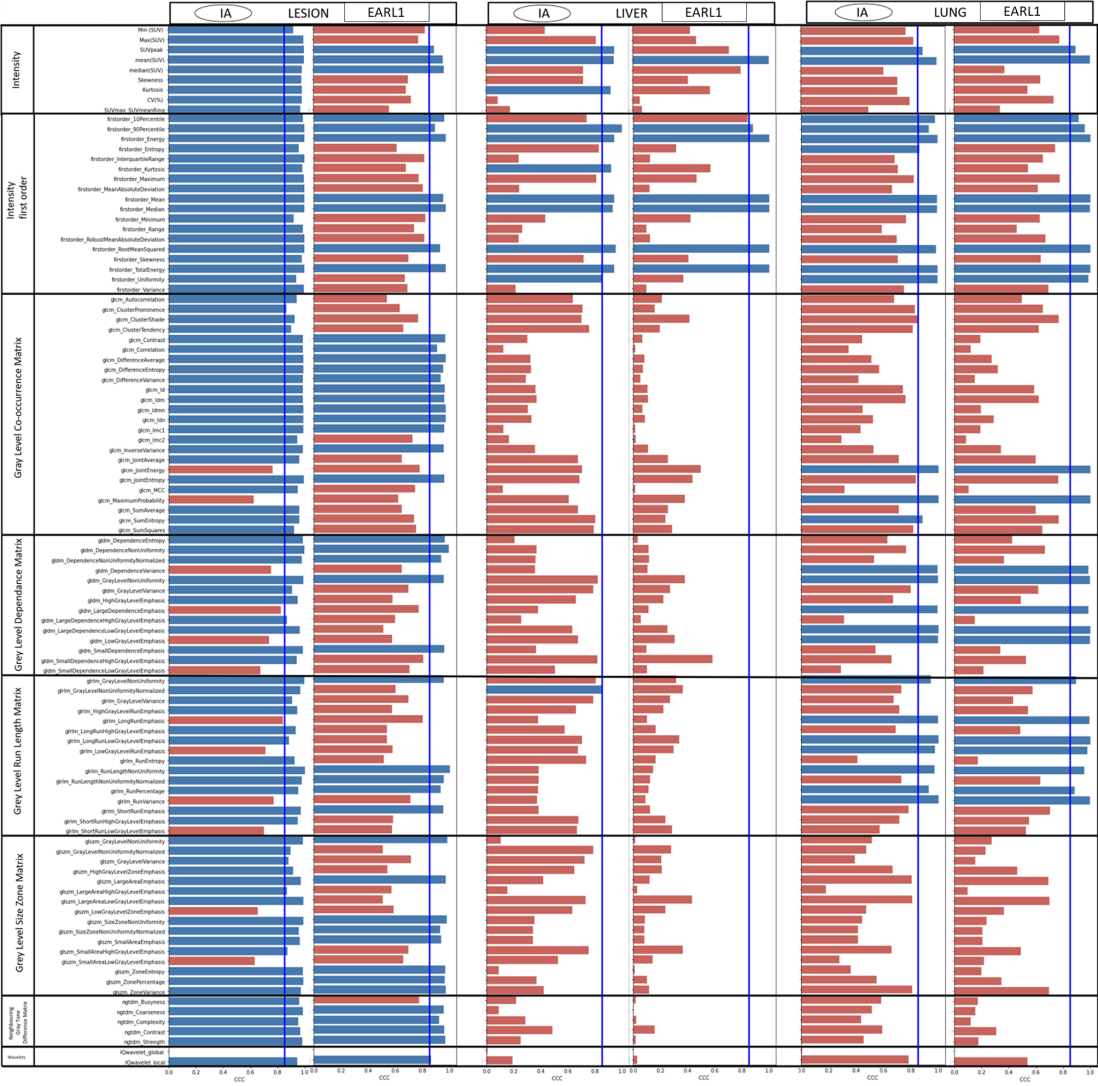
CCC of all the features from AI and EARL *versus* original images. A threshold is display by a line with a CCC = 0.85. Blue bar indicates a CCC≥0.85, red bar CCC < 0.85.

The concordance correlation coefficient (CCC) testifying of the stability of the features comparing denoised to original images is presented in **Figure 2**. In lesions, 90/104 (86.5%) with AI and 46/104 (44%) with EARL1 denoising stayed stable. All stable features in the EARL1 images were also stable in AI images. For the basic intensity class parameters, SUVpeak, SUVmean and SUVmedian kept a CCC≥0.85 in the two denoising approaches. SUVmax and SUVmin CCC values stayed stable for the AI denoised images in the lesions, but fell below the significant threshold for EARL1 images. The NGTDM features were less affected by both denoising methods. In the reference organs, for AI and EARL1 respectively, 12/104 (11.5%) and 7/104 (6.7%) in liver and 26/104 (25%) and 24/104 (23.1%) in lung had a CCC value at least of 0.85. The majority of the features in reference organs are less stable then in lesions for the two denoising methods. For the basic intensity parameters, SUV mean was overall stable for both denoising methods while SUV peak in both liver and lung for AI denoising, *versus* only in the lung for EARL1.

**Figure 3:**
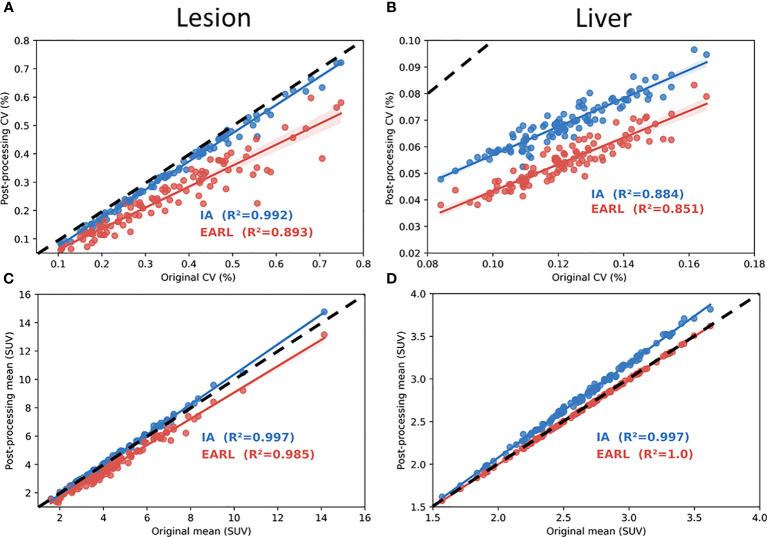
Coefficient of correlation plot with R^2^ value in lesions **(A, C)** and healthy liver **(B, D)**. **(A–D)** show respectively coefficient of variation (CV) and mean SUV calculated from AI and EARL1-like image in function of original images. Dotted line represents the identity line.

Concerning AI denoising, CV values in lesions before and after processing were very similar. In the lesions the values were slightly below and parallel to the identity line with R^2^ = 0.992. EARL1 showed a lower correlation and greater distance from the identity line(**Figure 3A**). In healthy liver (**Figure 3B**), the behavior was different. CV was reduced by a magnitude order of 2 for both denoising methods. With IA denoising, the points were also more scattered for liver (R^2^ = 0.884) than for lesions (R^2^ = 0.992). EARL1 denoising showed less differences (R^2^ = 0.851 *vs* 0.893). The SUV mean value displayed in **Figures 3C, D** showed high correlation in lesions as well as in the healthy tissue. In **Figure 3D** SUV mean in liver is not modified by a EARL1 gaussian postfilter (R^2^ = 1). Scatter plots of variable pairs for all the features are accessible in the supplementary materials.

**Figure 4:**
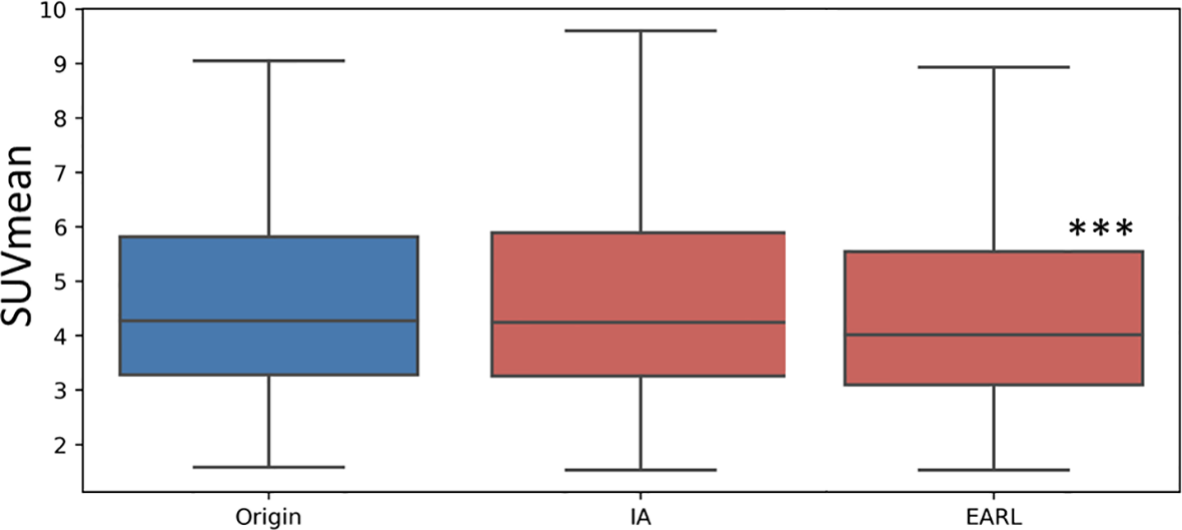
Box plot of the mean SUV value in lesion in Original, AI and EARL1-like images. The distribution difference between the original images and AI is not significant (p=0.06) while it is significant (p<0.001 ***) between original and EARL1-like images.

**Figure 4** testifies of the difference of SUV mean in lesions between AI and EARL1 denoising compared to the original images. AI denoising not significantly modified the SUV mean values with a p=0.06. EARL1 post filter led to a significantly lower mean SUV in lesions (p<0.001).

The results of the paired Wilcoxon signed rank test between original and AI denoised images are presented in **Table 2**. Almost all the features 79/100 (79%) were significantly different. Wavelets features were not studied. In the intensity class only 4/27 were not significantly different. SUV mean and median values were not significantly different between the AI denoised image and the original one. **Table 2** shows in blue the 18 features that had a CCC>0.85 and were not significantly different.

**Table 2:**
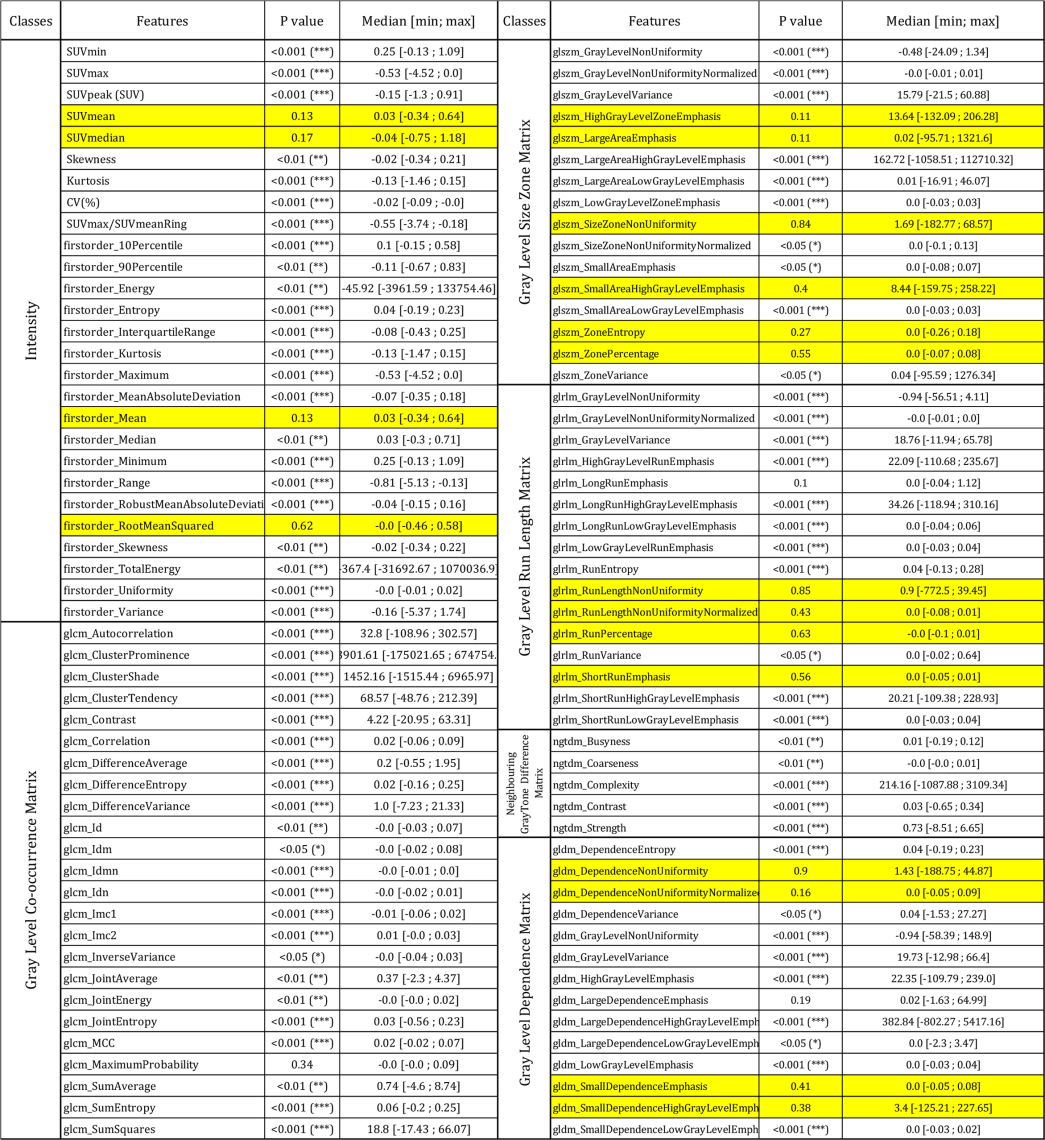
Result of Wilcoxon signed rank test of all the features between AI denoised and original images in lesions.

*p < 0.05, **p < 0.01 and ***p < 0.001. Yellow filling means that the features are not significantly different p>0.05 and have a CCC>0.85.

## Discussion

We evaluated the impact of AI denoising on the stability of radiomics features computed in FDG PET images, the standard being the clinical images. We also concurrently evaluated the effect of EARL1 gaussian filtering. To the best of our knowledge, it is the first clinical study on the impact of artificial intelligence denoising on PET radiomics.

Texture features used in radiomics models describe the pattern distribution of voxels and quantify intra-tumor heterogeneity in all 3 dimensions (4). 86.5% showed a stable behavior for intensity and radiomics classes. The stability criterium was based on a CCC≥0.85 (20). In lesions, values were significantly different in 71.1% of the features after AI denoising. An AI denoising approach like CNN seems to change the absolute values of most of the features but keep the correlation between them.

Advanced applications aim at the correlation of image features, like radiomics, with clinical endpoints (4, 23). Radiomics models derived from CT correlated with a prognostic value, overall survival in lung cancer patient (5). In baseline PET of locally advanced rectal cancer ^18^F-FDG PET/CT texture features provide strong independent predictors of survival in patients (24). These models are very promising however there are several pitfalls to overcome (25) such as study design, data acquisition, segmentation, features calculation and modeling by the radiomics community. This study allows a better understanding of the behavior of predictive models when an AI denoising method is employed. A predictive model based on this type of information can be built from MRI, PET, CT or a combination of image modalities.

Deep learning AI techniques have been used to perform denoising on PET images for example by generating a full-dose PET images from low-dose images (26) or to directly filter reconstructed PET images (27). We used an AI denoising approach based on DCNN (11). This approach seems to be able to reduce the acquisition time activity product by a factor of 2 to 4. We used it directly on the studied PET image without activity or time reduction because we want to characterize the effect of AI denoising while not compensating for count losses.

Denoising will be more and more used but may also generate pitfalls to build a radiomics predictive model as the 3d texture information may be modified. Studying the stability of features with a test-retest approach has been performed in PET (28). The number of features selected based on their stability was 71% (CCC>0.8) in PET NSCLC patients. In this study the stability of FDG PET radiomics features in lesions was 86.5% (CCC≥0.85) between AI denoised and original images. These values are at least of the same order of magnitude highlighting the performance of AI for denoising in PET imaging. As a consequence, a predictive model built on standard PET images could be transposed on AI denoised images, especially concerning the features we have shown as stable in this study. However, the threshold values will have to be recomputed. On the other hand, in healthy tissues as liver and lung most of the features were unstable. Stable features were even less frequent in the liver (11.5%) than in the lung (25%). The effect of denoising on these tissues seems more drastic than on lesions. We hypothesize that the AI algorithm recognizes similar healthy features and changes their intensity value and distributions. As a consequence, the ratio of the lesion over liver uptake should be transposed with care in clinical PET evaluation, as this ratio is altered for AI denoised versus original PET images.

The difference of behaviors in lesions and healthy tissue is one of the main advantages of AI based methods compared to an EARL1 gaussian post filter method. AI denoising maintains in the lesion the textural information and FDG uptake more stable while modifying healthy tissue. CV measures noise but also grey levels and is correlated to NECR/image quality in PET (29). As shown in **Figures 3A, B**, AI denoising had almost no effect on CV in lesions but reduced it in liver. On the contrary, EARL1 Gaussian postfilter reduced CV similarly in lesions and liver. Gaussian post filter will apply denoising accounting for neighbor all over the images whereas AI may be more selective in amplitude of denoising depending on noise vs non-noise components. The distribution of SUV mean in lesions has a different behavior between AI (paired t-test p=0.06) and EARL1 post filter (paired t-test p<0.001) compared to original images.

Interestingly EARL1 gaussian postfilter led to no modification of SUV mean values in liver (**Figure 3D**). It is mainly due to the increase of point spread function caused by the application of a gaussian postfilter. In a large homogenous area SUVmean was not modified while the noise (CV) was reduced. In smaller, more heterogeneous areas it will melt the grey levels of the different neighbors, lesions and healthy voxels (30). The modification of all the tissues in the image by the gaussian postfilter also appeared in **Figure 2**. Even in lesions only 44% of the features remained stable in EARL1 compatible versus original PET images.

In this analysis we tried to minimize the bias inherent of a radiomics workflow. We use pyradiomics which is mostly compatible with IBSI initiative. Each AI and EARL1 denoised images were extracted from the same images. The same VOI were used on all the series. One could however point out the use of the same contours for lesions in the 3 images as a possible study drawback. Re-segmentation of lesions on each image could have led to different contours and feature values. There is no gold standard for a segmentation method in PET radiomics. It remains also unclear to which extent this can affect radiomics values and predictive models (31). We chose a resampling of 64 bins instead of a fixed bin width (32) even if it showed a better reproducibility. As we directly compared images before and after denoising (minimum and maximum values of the image changed) resampling with a fixed bin width could lead to a different number of bins just due to noise reduction and not to texture based information. In a future work we would apply the same methodology with bin width resampling to strengthen our outcome. We didn’ t split the data into training, validation and test cohorts in this study due to the relatively small number of patients and lesions (33). A test-retest radiomics study on patient in CT showed that 446/542 features had a higher CCC for patients with lung cancer than for those with rectal cancer (34). Our study was based on 113 patients, which is a small number. Pooling however different primary malignancies and lesions’ nature and size might have helped to reduce overfitting. The main next challenge will be to validate our findings on different and heterogenous patient cohorts and other PET protocols and systems. It might be very risky to apply the same selection of features on other PET or even MRI or CT systems (25). Also, the mechanism of AI denoising recognizing successfully non noise *versus* noise components has to be further investigated on other camera types and PET protocols.

Numerical PET/CT’s have a better spatial and temporal resolution leading to a more contrasted activity distribution in lesions than analog systems (35). As this study was carried out on a digital PET/CT we can expect that it will have been more sensitive to variations in texture compared to one on an analog system.

## Conclusion

Applying an AI, CNN denoising on FDG PET images maintains most of the lesion’s texture information in contrast to a EARL1-compatible Gaussian postfilter. The predictive texture features of a trained model could be transposed, however with an adapted threshold. Artificial intelligence in PET is a very promising approach as it adapts the denoising for noise *versus* non-noise components preserving information where it should.

## Data Availability Statement

All the data and the python code of the analysis are available on https://github.com/AurelienCD/RadiomicsIA_PET_Depository_Manuscript-ID-692973.

## Ethics Statement

Ethical review and approval was not required for the study on human participants in accordance with the local legislation and institutional requirements. Written informed consent for participation was not required for this study in accordance with the national legislation and the institutional requirements.

## Author Contributions

CJ has designed the study, computed the radiomics and written the majority of the manuscript. KW included study patients, performed image analysis including segmentation, wrote manuscript parts and substantially revised it. AC-D has computed the statistics and written manusript sections. AL gave feedback on the AI algorithm. AB helped to design the study. SB gave advice and feedback on the study. All authors contributed to the article and approved the submitted version.

## Conflict of Interest

The authors declare that the research was conducted in the absence of any commercial or financial relationships that could be construed as a potential conflict of interest.

## Publisher’s Note

All claims expressed in this article are solely those of the authors and do not necessarily represent those of their affiliated organizations, or those of the publisher, the editors and the reviewers. Any product that may be evaluated in this article, or claim that may be made by its manufacturer, is not guaranteed or endorsed by the publisher.
