# Supplementary material for: The Impact of Artificial Intelligence CNN Based Denoising on FDG PET Radiomics
Source: Front Oncol. 2021 Aug 24;11:692973. doi: 10.3389/fonc.2021.692973 (PMC8421788; doi:10.3389/fonc.2021.692973)
Supplement: Supplementary file 1 [file DataSheet_1.docx]

Supplementary Material

Annexe 1: Extraction of radiomics python code

# coding: utf-8

import unittest

from slicer.ScriptedLoadableModule import *

import logging

from __main__ import vtk, qt, ctk, slicer

from math import *

import numpy as np

from vtk.util import numpy_support

import SimpleITK as sitk

import sitkUtils as su

import time

import codecs

import datetime

import vtkSegmentationCorePython as vtkSegmentationCore

import pydicom

import sys, time, os

import pywt # https://github.com/PyWavelets/pywt/blob/master/pywt/_multilevel.py

import pandas as pd

from pandas import ExcelWriter

from pandas import ExcelFile

import numpy as np

############################si il manque une biblio exemple scikit #################################

#slicer.util.pip_install("scikit-image")

#########################################################

image_directory = ""

label_directory =""

adresse_save_result=""

def cropImagefctLabel(image, LowerBondingBox, UpperBondingBox ):

#crop=sitk.CropImageFilter()

image_cropper=sitk.Crop(image, LowerBondingBox, UpperBondingBox )

return image_cropper

def getSec(s):

b =int(s[0:2]) *3600 + int(s[2:4])*60 + int(s[5:6])

return b

#import radiomics

from radiomics import featureextractor # This module is used for interaction with pyradiomics

def radiomics_extraction(image,label):

extractor = featureextractor.RadiomicsFeatureExtractor()

# First define the parameters

params = {}

params['binWidth'] = 64 ##binCount

params['sigma'] = [1] #[1, 2, 3]

params['verbose'] = True

params['correctMask']=True

# Instantiate the extractor

extractor = featureextractor.RadiomicsFeatureExtractor(**params) # ** 'unpacks' the dictionary in the function call

#########################################################################################################################

#option supplementaire:

# Enable a filter (in addition to the 'Original' filter already enabled)

#extractor.enableInputImageByName('LoG')

#print ""

#print "Enabled filters:\n\t", extractor.inputImages

# Disable all feature classes, save firstorder

#extractor.disableAllFeatures()

#extractor.enableFeatureClassByName('firstorder')

#print ""

#print "Enabled features:\n\t", extraenabledFeatures

#######################ctor.enabledFeatures

# Specify some additional features in the GLCM feature class

#extractor.enableFeaturesByName(glcm=['Autocorrelation', 'Homogeneity1', 'SumSquares'])

#print ""

#print "Enabled features:\n\t", extractor.#####################################################################################################

####################################traitement ###########################

result = extractor.execute(image, label) #segment base

##################################traitement voxel base##############

#result = extractor.execute(image, label,voxelBased=True)

#print "Result type:", type(result) # result is returned in a Python ordered dictionary

#print ""

#print "Calculated features"

#for key, value in result.iteritems():

# print "\t", key, ":", value

return result

#############################################################################

#######################ondelette analysis####################################

def SpatialFrequencyOptim(matrix):

sq_diff = 0.0

size=matrix.shape

dim=len(size)

#arr[arr > 255] = x

#boolArr = matrix>0.05

#matrix = matrix[boolArr] # on ne prend pas en compte les voxels en dehors du body

for i in range(dim): #iterate over all image dimensions

slc1 = [slice(None)]*dim

slc1[i] = slice(0,size[i]-1)

slc2 = [slice(None)]*dim

slc2[i] = slice(1,size[i])

sq_diff+= np.sum((matrix[tuple(slc2)]- matrix[tuple(slc1)])**2)

return sq_diff/np.prod(size)

def Ondelette_raconte_global(image):

NumpyImage=sitk.GetArrayFromImage(image)

max_lev = 2 # how many levels of decomposition to draw

c = pywt.wavedecn(NumpyImage, 'db2', mode='zero', level=max_lev) #voir https://pywavelets.readthedocs.io/en/latest/ref/nd-dwt-and-idwt.html#pywt.wavedecn

c_arr,c_slices= pywt.coeffs_to_array(c, padding=0, axes=None)

ddd=c_arr[c_slices[2]['ddd']]

aaa=c_arr[c_slices[0]]

#ddd=sitk.GetImageFromArray(c_arr[c_slices[2]['ddd']]) #details

#aaa=sitk.GetImageFromArray(c_arr[c_slices[0]]) #average

IndiceQualite=SpatialFrequencyOptim(ddd)/SpatialFrequencyOptim(aaa)

return IndiceQualite

def Ondelette_raconte_local(image):

NumpyImage=sitk.GetArrayFromImage(image)

max_lev = 1 # how many levels of decomposition to draw

c = pywt.wavedecn(NumpyImage, 'db2', mode='periodic', level=max_lev) #voir https://pywavelets.readthedocs.io/en/latest/ref/nd-dwt-and-idwt.html#pywt.wavedecn

c_arr,c_slices= pywt.coeffs_to_array(c, padding=0, axes=None)

ddd=c_arr[c_slices[1]['ddd']]

aaa=c_arr[c_slices[0]]

#ddd=sitk.GetImageFromArray(c_arr[c_slices[1]['ddd']]) #details

#aaa=sitk.GetImageFromArray(c_arr[c_slices[0]]) #average

IndiceQualite=SpatialFrequencyOptim(ddd)/SpatialFrequencyOptim(aaa)

return IndiceQualite

#################################################################################################

##################################################################################################

def MatriceDeResultat(nom ,label, stat_filter ,result, IQwavelet_global,IQwavelet_local, Data,stat_filter_ring):

###ecriture des metada pour correspondant au patient###

N_result=13+len(result.values())

ArrayOfResult=np.zeros(N_result)

###ecriture des metada pour correspondant au patient###

ArrayOfResult[0]=str(nom)

ArrayOfResult[1]=str(label)

ArrayOfResult[2]=str(stat_filter.GetMinimum(label))

ArrayOfResult[3]=str(stat_filter.GetMaximum(label))

ArrayOfResult[4]=str(stat_filter.GetMean(label))

ArrayOfResult[5]=str(stat_filter.GetMedian(label))

ArrayOfResult[6]=str(stat_filter.GetSkewness(label))

ArrayOfResult[7]=str(stat_filter.GetKurtosis(label))

ArrayOfResult[8]=str(stat_filter.GetPerimeterOnBorderRatio(label))

ArrayOfResult[9]=str(stat_filter.GetStandardDeviation(label)/stat_filter.GetMean(label))

ArrayOfResult[10]=str(stat_filter.GetMaximum(label)/stat_filter_ring.GetMean(10))

ArrayOfResult[11]=str(IQwavelet_global)

ArrayOfResult[12]=str(IQwavelet_local)

N=12

for value in result.values():

N=N+1

ArrayOfResult[N]=str(value)

Data=np.append(Data,ArrayOfResult)

print(" Analyse:ok")

return Data

########################################################################################

########################################################################################

def main_image_Traitement(image,label_template,nom,f,firsttime,Data):

###################extration des résulats standard################

stat_filter=sitk.LabelIntensityStatisticsImageFilter()

stat_filter_ring=sitk.LabelIntensityStatisticsImageFilter()

stat_filter.Execute(label_template,image)

IQwavelet_global=Ondelette_raconte_global(image)

print( "main_image_Traitement: Ondelettes global")

Data= np.empty([0, 2]) ###########intialize an empty 2d array

for label in stat_filter.GetLabels():

if (IsTextureAnalysis==True or IsWaveletDecompose==True) :

####crop pour acceleration############################################

label_select=sitk.BinaryThreshold(label_template, label, label, 1,0)

stats= sitk.LabelIntensityStatisticsImageFilter()

stats.Execute(label_select,image)

delta=5 #extention du label pour eviter les problemes aux bords + pour anneau de background

LowerBondingBox=[stats.GetBoundingBox(1)[0]-delta,stats.GetBoundingBox(1)[1]-delta,stats.GetBoundingBox(1)[2]-delta]

UpperBondingBox=[image.GetSize()[0]-(stats.GetBoundingBox(1)[0]+stats.GetBoundingBox(1)[3]+delta),image.GetSize()[1]-(stats.GetBoundingBox(1)[1]+stats.GetBoundingBox(1)[4]+delta),image.GetSize()[2]-(stats.GetBoundingBox(1)[2]+stats.GetBoundingBox(1)[5]+delta)]

image_select=cropImagefctLabel(image, LowerBondingBox, UpperBondingBox )

label_select=cropImagefctLabel(label_select, LowerBondingBox, UpperBondingBox )

#su.PushToSlicer(image_select,"ii",1)

#su.PushToSlicer(label_select,"ll",2)

#print "main: image dans espace TDM crop ok"

print(" main_image_Traitement: Crop")

######################## extraction resultats de l'analyse de texture#######################

result=radiomics_extraction(image_select,label_select)

print(" main_image_Traitement: Radiomics")

##########################################extraction indice qualité ondelette

IQwavelet_local=Ondelette_raconte_local(image_select)

print(" main_image_Traitement: Ondelettes local")

#creation d'un anneau autour de la lesion:

############# enlever les trous dans le volume cible: morphological closing surface#####

labelmap=10-10*label_select

stat_filter_ring.Execute(labelmap,image_select)

#labelmap=sitk.BinaryErode(labelmap)

############# enlever les trous dans fond: morphological closing surface#####

#labelmap=sitk.BinaryErode(labelmap)

#labelmap=sitk.BinaryDilate(labelmap)

############écriture de la premiére ligne

if firsttime==0: #write for the first time all the description value

firsttime=1

N_Column=13+len(result.keys())

NameColumn=np.zeros(N_Column)

NameColumn[0]=str(PatientName)

NameColumn[1]=str(labels)

NameColumn[2]=str(Min (SUV))

NameColumn[3]=str(Max(SUV))

NameColumn[4]=str(mean(SUV))

NameColumn[5]=str(median(SUV))

NameColumn[6]=str( Skewness)

NameColumn[7]=str(Kurtosis)

NameColumn[8]=str(PerimeterOnBorderRatio)

NameColumn[9]=str("CV(%)")

NameColumn[10]=str("SUVmax/backgroundmean")

NameColumn[11]=str(IQwavelet_global)

NameColumn[12]=str(IQwavelet_local)

N=12

for key in result.keys():

N=N+1

NameColumn[N]=str(key)

Data=MatriceDeResultat(nom ,label, stat_filter ,result, IQwavelet_global,IQwavelet_local,Data,stat_filter_ring)

df=pd.DataFrame(Data,columns=NameColumn )

writer = ExcelWriter(adresse_save_result)

df.to_excel(writer,'RadiomicsEtCompany',index=False)

writer.save()

def main(image_directory,label_directory, adresse_save_result, IsTextureAnalysis, IsWaveletDecompose):

timeInit = time.time()

Nimageouverte=0

Nimagetraitees=0

firsttime=0 #for printing the radiomics key on the first line

f = open(adresse_save_result, 'w') #open the file for saving result

############# lecture des fichiers image ###########

for path, dirs, files in os.walk(image_directory):

for file in files:

Nimageouverte=Nimageouverte+1

try:

image_filepath = os.path.join(image_directory, file)

image = sitk.ReadImage(image_filepath)

filename, ext = os.path.splitext(file)

filename=filename+"-label.nrrd"

label_filepath = os.path.join(label_directory, filename)

label = sitk.ReadImage(label_filepath)

#su.PushToSlicer(image,"Itest",1)

#su.PushToSlicer(label,"Ltest",2)

timeRMR1 = time.time()

Nimagetraitees=Nimagetraitees+1

nom=str(file)

print("main :"+str(nom))

image= sitk.Cast(image, sitk.sitkFloat64)

#su.PushToSlicer(img, "image_"+series_ID,1) #test de rapatriement

main_image_Traitement(image,label,nom,f,firsttime)

firsttime=1 #just write the first ligne one time

timeRMR2 = time.time()

TimeForrunFunctionRMR2 = timeRMR2 - timeRMR1

print(u"La fonction de traitement s'est executée en " + str(TimeForrunFunctionRMR2) +" secondes")

print("\n")

except RuntimeError:

print ("--> Probleme avec l'importation et/ou le triatement d'image")

if firsttime!=0:

f.close()

print("\n")

print("Nombre d'image total lue:"+str(Nimageouverte)+"\n")

print("Nombre d'image total traité:"+str(Nimagetraitees)+"\n" )

timefinal = time.time()

TimeTotal = timefinal - timeInit

print(u"Le traitement de l'ensemble des données c'est executée en " + str(TimeTotal) +" secondes")

##################################Execution du code############################################

###############################################################################################

main(image_directory,label_directory, adresse_save_result, IsTextureAnalysis, IsWaveletDecompose)

**
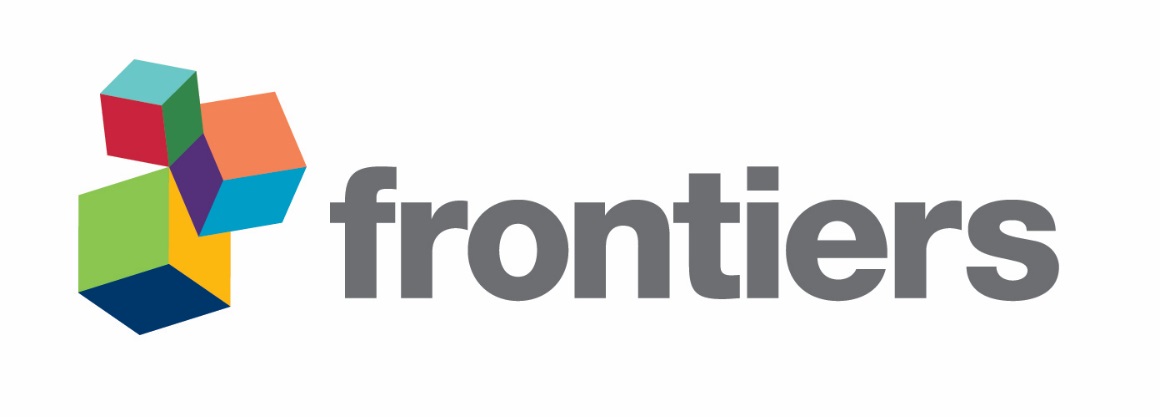
**

**Supplementary Figure 1.** The figure legends are required to have the same font as the main text, 12 point normal Times New Roman, single spaced. Please use a single paragraph for each legend and prepare the figures keeping in mind the PDF layout.
