# Supplementary figures and images for: The Impact of Artificial Intelligence CNN Based Denoising on FDG PET Radiomics
Source: Front Oncol. 2021 Aug 24;11:692973. doi: 10.3389/fonc.2021.692973 (PMC8421788; doi:10.3389/fonc.2021.692973)

## Slide 1
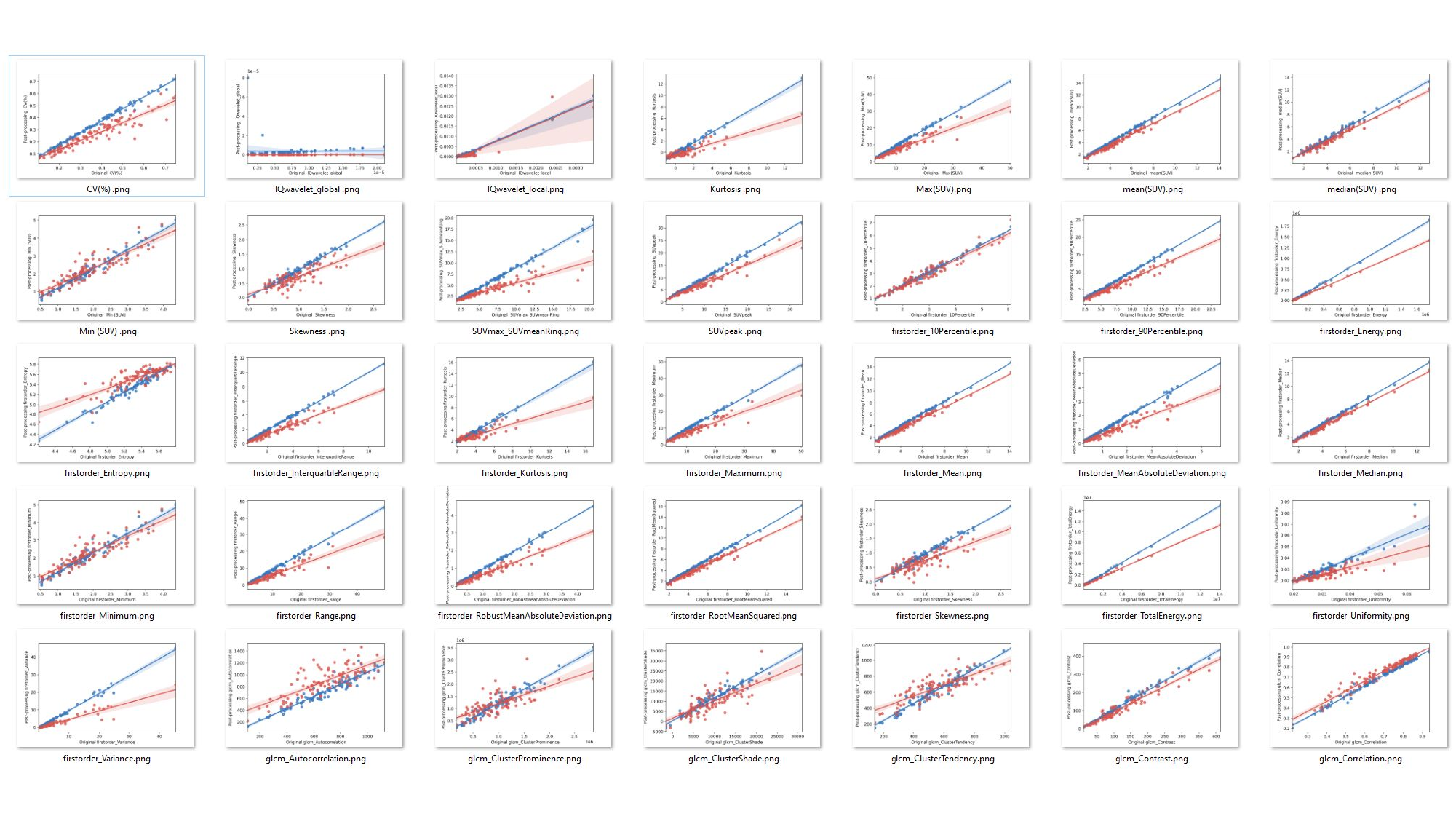

## Slide 2
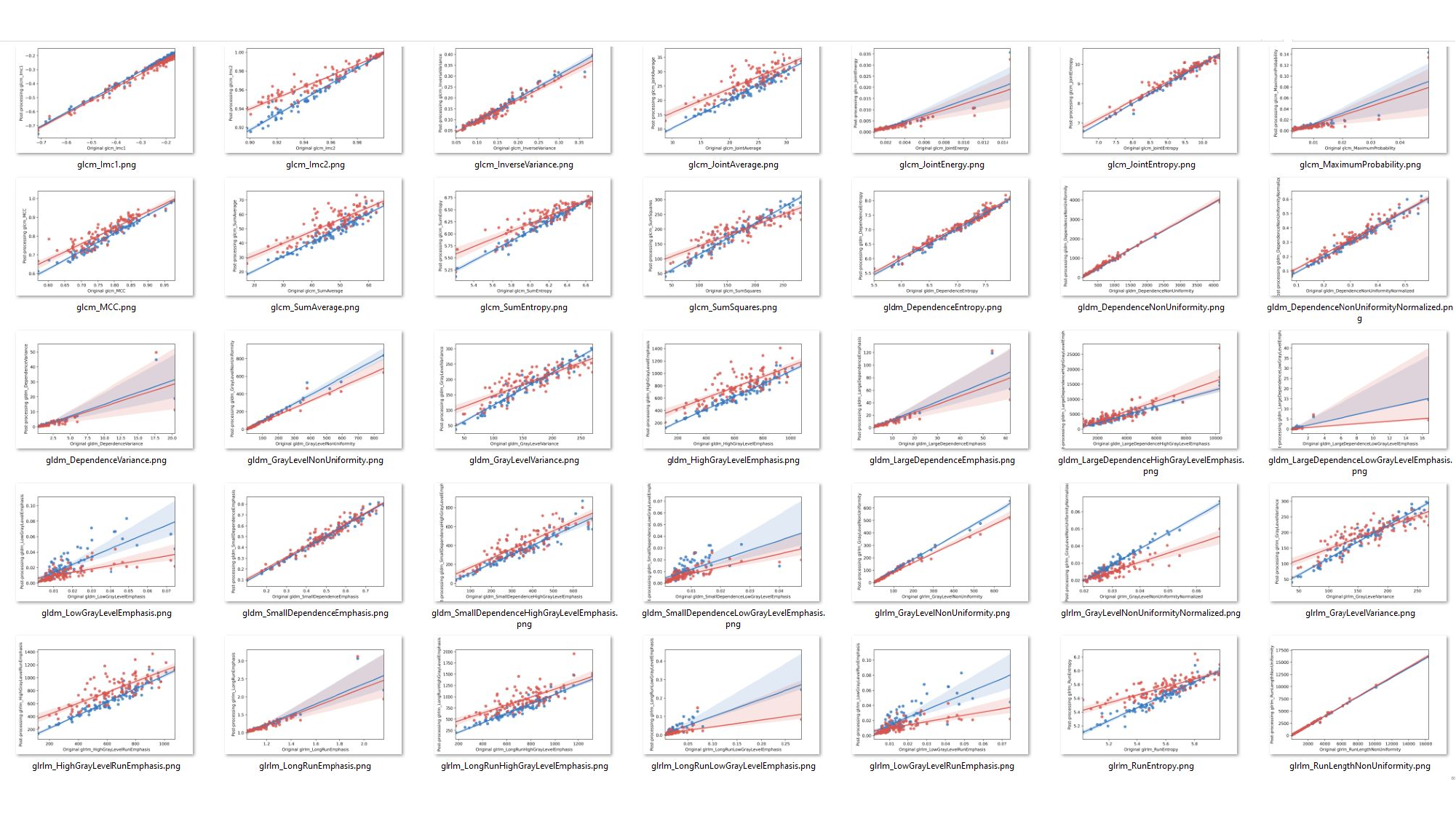

## Slide 3
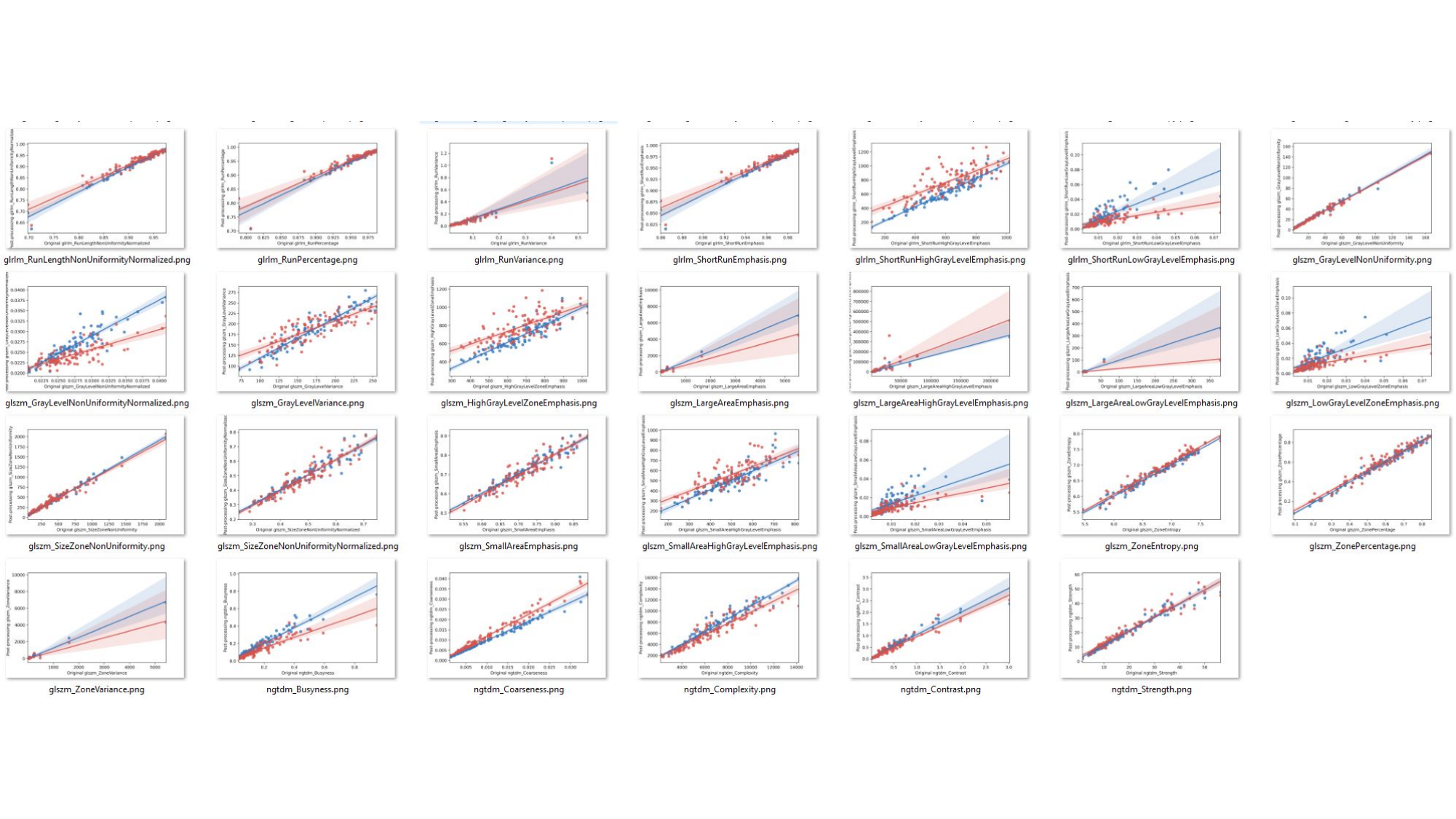

Supplement: Supplementary file 2 [file Presentation_1.pptx]
